# Evaluating the Impact of Retroperitoneal Lymphadenectomy in Terms of Morbidity and Survival in Advanced Epithelial Ovarian Cancer

**DOI:** 10.7759/cureus.92819

**Published:** 2025-09-21

**Authors:** Mukurdipi Ray, Bittu Bhukkal

**Affiliations:** 1 Surgical Oncology, All India Institute of Medical Sciences, New Delhi, New Delhi, IND

**Keywords:** carcinoma ovary, ivc injury, lymphadenectomy, lymphatic leak, retroperitoneal lymphadenectomy, rplnd

## Abstract

Background

This study aims to assess the role of routine systematic retroperitoneal lymphadenectomy (RPLND) in managing advanced ovarian cancer while maintaining acceptable levels of morbidity and without mortality associated with the procedure. However, concerns have emerged regarding its insufficiency in delivering survival benefits.

Method

A retrospective analysis of prospectively maintained electronic records from 2012 to 2023 was conducted at our tertiary referral center among 362 cases of advanced ovarian cancer. Patients with International Federation of Gynecology and Obstetrics (FIGO) stages III and IV cancer underwent optimal cytoreductive surgery (CRS) with standard upfront and interval procedures, including systematic RPLND, and selected cases underwent hyperthermic intraperitoneal chemotherapy (HIPEC). We analyzed RPLND-associated morbidity, encompassing both intraoperative and postoperative complications.

Result

The mean age of the patients was 55.2 years. The lymph nodes were found to be positive in 215 patients (59.39%), and the positivity rate was higher in those who underwent upfront surgery (n=139; 64.65%), as compared to those who received neoadjuvant chemotherapy (NACT; n=76; 35.35%). RPLND-related post-operative complications were vascular: inferior vena cava (IVC) injury (n=10; 2.76%), aortic injury (n=6; 1.6%), and lymphatic leaks in the postoperative period (n=21; 5.80%) patients. Out of these 21 patients, some developed lymphatic ascites (n=13; 3.59%), chylous ascites (n=8; 2.4%), and postoperative ileus (n=12; 3.32%). Recurrence was reported 104 patients (39.69%) during the follow-up period. For a median follow-up of 58.5 months, the median disease-free survival (DFS) was 32.3 months. The overall five-year survival in our patients was 48.9% (95% CI 35.5-61).

Conclusion

RPLND in advanced ovarian cancer was feasible, revealed high nodal positivity (>50%), and was associated with manageable morbidity without mortality. It facilitates precise disease staging and helps achieve optimal cytoreduction.

## Introduction

Ovarian cancer (OC) is the most lethal gynecological cancer worldwide, and the median age at diagnosis is around 63 years in most developed countries [1]. Nearly three-quarters of new cases are diagnosed at advanced stages [2]. OC is the eighth most common cancer and ranks eighth in cancer-related deaths globally. The standard treatment for advanced OC is cytoreductive surgery (CRS) followed by systemic chemotherapy. The surgical treatment of OC aims to achieve maximal cytoreduction [3]. According to the current concept, CRS encompasses the removal of all macroscopic residual disease; however, the routine removal of pelvic and retroperitoneal lymph nodes is not standardised as a part of cytoreduction. This theoretically violates the principle of CRS from a scientific point of view. Lymph node status is an important prognostic factor in patients with OC. The retroperitoneal lymphatic spread has been a common feature, especially in patients with advanced OC. The rate of lymph node metastasis is about 20-41%, which can reach up to 50-80% in advanced patients (International Federation of Gynecology and Obstetrics (FIGO) stages III-IV). Even with post-neoadjuvant chemotherapy (NACT), around 50-60% of nodes are positive for malignancy [4].

The National Comprehensive Cancer Network (NCCN) recommends that systematic retroperitoneal lymphadenectomy or RPLND (including pelvic and paraaortic lymphadenectomy) should be included in the primary surgery in early-stage patients with OC. However, in advanced stages, the benefit of lymphadenectomy is still controversial, though some retrospective studies showed an improved prognosis [4-6]. Recently, contrary to the previous study, the Lymphadenectomy in Ovarian Neoplasms (LION) study, which was a well-designed randomised controlled trial, did not report any survival advantage for systematic lymphadenectomy in patients without bulky lymphadenopathy. In addition, RPLND may increase intraoperative and postoperative complications, such as bleeding, vascular injury, lymphocyst, infection, intestinal fistula, and chylous fistula [7]. Given the above controversial issue, is RPLND associated with more postoperative complications with no survival benefit? We performed a retrospective analysis to investigate the role of RPLND in terms of complications.

## Materials and methods

We conducted a retrospective analysis based on prospectively maintained electronic records of peritoneal surface malignancies from 2012 to 2023 at the Department of Surgical Oncology, All India Institute of Medical Sciences (AIIMS), New Delhi, India. The majority of OC cases in our institute manifested at advanced stages, specifically FIGO stages III and IV. As a tertiary referral centre for cancer care, over 90% of OC cases are at an advanced stage. Between 2012 and 2023, a total of 362 patients with advanced OC who underwent surgical treatment at our institute were analysed. Patients who underwent incomplete surgery elsewhere, those with prior pelvic or retroperitoneal surgeries, uncertain treatment histories, irregular treatment patterns, or a history of pelvic irradiation were excluded from the analysis.

Preoperative assessment included contrast-enhanced computed tomography scans (CECT) of the abdomen and pelvis, as well as the measurement of tumour markers cancer antigen 125 (CA-125), carcinoembryonic antigen (CEA), and carbohydrate antigen 19-9 (CA 19.9) in all cases. Based on discussions in our multidisciplinary tumour board, patients with poor performance status, multiple comorbidities, CA-125 levels exceeding 2000, high radiologic peritoneal cancer index (PCI), and involvement of the liver or spleen parenchyma were considered for NACT followed by interval CRS. Other patients underwent three to four weeks of prehabilitation before primary CRS.

Surgical procedures included standard primary cytoreduction steps such as peritoneal washings, total abdominal hysterectomy with bilateral salpingo-oophorectomy, total omentectomy, and multiple quadrant peritoneal biopsies. Bowel resections, disease-specific peritonectomy, and total peritonectomy were performed selectively to achieve complete cytoreduction (CC-0 or CC-1). Routine dissection of the retroperitoneal and bilateral pelvic lymph nodes was performed in all cases. Optimal cytoreduction was defined as no residual tumour or residual tumour size equal to or less than 2.5 mm, as selected cases underwent hyperthermic intraperitoneal chemotherapy (HIPEC).

All patients received adjuvant chemotherapy following CRS and were followed up regularly with history and clinical examination every three months, along with CA 125 measurements and imaging every six months.

Patient and disease characteristics were assessed as part of the analysis. Rates of optimal cytoreduction and overall survival (OS) were evaluated across cohorts stratified by lymph node status (positive vs. negative). Survival outcomes were analyzed using the Kaplan-Meier method, implemented in STATA software (version 14.0; StataCorp, College Station, TX, USA). OS was defined as the time interval from the initial diagnosis to death from any cause.

## Results

Our study analysed patients with OC who underwent retroperitoneal lymph node dissection (n=362). The mean age of the patients was 55.2 years (range 19-67). Nearly 80.9% of them had an Eastern Cooperative Oncology Group (ECOG) performance status of one, and 19.1% had a performance status (PS) of two. A mean of 25 nodes was dissected per case. The mean duration of surgery was 389 minutes (range: 200-550 min), and the mean blood loss was 403 ml (range: 100-800 ml). Moreover, some underwent bowel resection because of advanced disease (n=48), and stoma formation (n=20). The lymph nodes were found to be positive in more than half the patients (n=215; 59.39%), and the positivity rate was higher in patients who underwent upfront surgery (n=139; 64.65%), as compared to those who received NACT (n=76; 35.35%). The majority of patients (n=122; 56.74%) had positive lymph nodes in both the pelvic and retroperitoneal groups, whereas some had isolated pelvic nodes (n=49; 22.79%) and positive retroperitoneal nodes (n=44; 20.46%). The mean ICU stay was 1.6 days, whereas the mean hospital stay was 8.5 days. 

RPLND-related post-operative complications included vascular injury (n=16; 4.4%), IVC injury (n=10; 2.76%), and aortic injury (n=6; 1.6%) as shown in Table 1.

**Table 1:** Complications related to retroperitoneal lymphadenectomy (RPLND) IVC: Inferior vena cava

Complication		Number of patients	Percentage (%)
IVC injury		10	2.76
Aorta injury		6	1.65
Lymphatic ascites		13	3.59
Chylous ascites		8	2.20
Lymphoedema		7	2
Lymphatic cyst		5	1.40
Prolonged ileus	12		3.31

Some patients had lymphatic leaks in the postoperative period (n=21; 5.80%), and they were diagnosed at or after three days in a majority of cases.

All patients were managed conservatively with dietary modification and the drain was removed after a mean duration of 9.2 days, none of them required surgical intervention. Intra-abdominal collection (n=9) was managed conservatively by draining of collection through pigtail catheters, some patients had a bowel leak (n=7), and one had a biliary fistula (n=1). Six patients underwent relaparotomy and one patient underwent endoscopic retrograde cholangiopancreatography (ERCP) and stenting. Some patients underwent readmission (n=26; 7.18%), as shown in Table 2.

At a median follow-up of 58.5 months, the median DFS was 32.3 months. The five-year OS in our patients was 48.9% (95% Cl 35.5-61).

**Table 2:** Complications related to surgery SSI: Surgical site infection; DVT: Deep vein thrombosis.

Complication	Number of patients	Percentage (%)
SSI	20	5.52
Intestinal fistula	6	1.65
Relaparotomy	6	1.65
Re-admission	26	7.20
DVT	7	2

## Discussion

Pelvic and paraaortic lymphadenectomy is frequently performed in cases of OC and constitutes a significant aspect of the surgical staging process. In the literature, positive lymph nodes were found in 30 to 75% of patients [8,9]. In our present study, the lymph node positivity rate was 59.39%. Isolated RPLND positivity was present in 20.46% of patients.

As a hypothesis, nodal metastasis might exhibit reduced sensitivity to chemotherapy and resistance to chemotherapy owing to compromised blood circulation, suggesting that lymphadenectomy could potentially enhance survival outcomes [10]. In a randomized clinical trial of 427 patients with optimally debulked advanced OC, Panici et al. compared systematic aortic and pelvic lymphadenectomy with resection of bulky nodes only. The prevalence of lymph node involvement was significantly higher in the systematic lymphadenectomy arm (70%) compared to the control arm (42%) [11]. Similarly, in a randomized trial by Maggioni et al. involving patients with epithelial OC, systematic lymph node dissection was associated with a higher incidence of histologically-confirmed nodal metastasis compared to macroscopic lymph node resection (22% vs. 9% in early-stage disease; 70% vs. 42% in advanced-stage disease) [12]. In the present analysis, the overall nodal positivity was 59.39%, with 64.65% in upfront cases and 35.35% in post-NACT cases.

Eoh et al. [9] demonstrated the therapeutic role of lymph node dissection with better prognosis in advanced OC with optimal cytoreduced surgery. In general, lymphadenectomy is recommended in cases where radiological evidence indicates nodal involvement. In 1995, Spirtos et al. demonstrated the benefit of removing bulky lymph nodes, but there was no clear benefit in resecting clinically negative lymph nodes [13]. The first randomised prospective trial by Panici et al. [11] showed that patients who underwent systematic lymphadenectomy had a longer progression-free survival compared to those who did not (29.4 months vs. 22.4 months, a difference of seven months) and experienced lower recurrence rates. However, there was no significant difference in OS between the two groups (58.7 months vs. 56.3 months). In 2010, Du Bois et al. analysed three randomised controlled trials including 1,904 patients and demonstrated that lymphadenectomy may be beneficial in the setting of complete cytoreduction. The five-year survival rate was significantly higher in patients who underwent lymph node resection compared to those without lymph node resection (67.4% vs. 59.2%, P=0.0166) [14]. In 2012, Chang et al., in a retrospective study (n=189), demonstrated the survival benefit of systematic lymphadenectomy in patients with stage II OC without any grossly visible disease [15]. However, in the LION trial, a randomized controlled trial published in 2019 by Harter et al. [16], patients with no suspicious lymph node (n=647; FIGO stage IIB-IV) were randomized at the end of cytoreductive surgery to the lymphadenectomy (n=323) and no-lymphadenectomy (n=324) groups. They demonstrated systematic pelvic and paraaortic lymphadenectomy in these patients was not associated with longer survival than no lymphadenectomy and was associated with a higher incidence of postoperative complications.

Various studies showed that chemotherapy alone does not eradicate involved nodes, leading to the persistence of positive nodes that may serve as potential sites for relapse, essentially acting as a "safe haven" [17]. Consequently, nodal involvement following chemotherapy could serve as an indicator of disease aggressiveness or potentially herald impending relapse. Several studies have demonstrated the potential survival benefit associated with systematic lymphadenectomy in post-chemotherapy scenarios. In a Surveillance, Epidemiology, and End Results (SEER) registry data analyzed by Yin and Wang [18], patients (n=10,184), patients underwent debulking surgery with RPLND (n=5472), or debulking surgery alone (n=4712). This study demonstrated that RPLND improved the survival outcome during debulking surgery in patients with advanced epithelial OC. The OS rate in our study at five years was 48.9% (95% Cl 35.5-61), which is comparable with the previous retrospective studies on RPLND [12,18].

Retroperitoneal lymph nodes do not come into the picture of survival in OC patients who die because of cancer cachexia or intestinal failure before lymph node enlargement. Any disease involving nodes indirectly destroys the immune system of the body and hampers survival, which is difficult to prove. That is why this controversy persists. We strongly objected to the result of the LION trial due to its failure to achieve a complete CRS (CC score=0), as leaving nodes behind contradicts the core principle of staging as well as HIPEC [19].

Several limitations should be acknowledged in our study. First, it was retrospective in nature and conducted at a single center. Second, the study population consisted exclusively of patients with advanced-stage epithelial OC, and we did not include a comparison with patients who did not undergo lymphadenectomy to evaluate complication rates. Nevertheless, our findings still provide valuable evidence supporting the role of lymphadenectomy in advanced OC.

## Conclusions

Over the past several decades, various researchers have scrutinized the utility of lymphadenectomy for both staging and therapeutic purposes, but no study has been able to resolve the controversy. In our experience, RPLND has been associated with improved recurrence-free survival and OS outcomes in advanced OC with acceptable morbidity and without mortality. RPLND helps to determine accurate staging and supports CC-0 resection, which leads to better survival. We strongly recommend RPLND in all cases of advanced OC.
